# A two-question tool to assess the risk of repeated falls in the elderly

**DOI:** 10.1371/journal.pone.0176703

**Published:** 2017-05-10

**Authors:** Alejandro Rodríguez-Molinero, César Gálvez-Barrón, Leire Narvaiza, Antonio Miñarro, Jorge Ruiz, Esther Valldosera, Natalia Gonzalo, Thalia Ng, María Jesús Sanguino, Antonio Yuste

**Affiliations:** 1 Consorci Sanitari del Garraf, Fundació Privada Sant Antoni Abat, Vilanova i la Geltrú, Barcelona, Spain; 2 Parc Sanitari Sant Joan de Déu, Centre de Salut Mental del Garraf, Vilanova i la Geltrú, Barcelona, Spain; 3 Department of Genetics, Microbiology and Statistics, Faculty of Biology, University of Barcelona, Barcelona, Spain; University of L'Aquila, ITALY

## Abstract

**Introduction:**

Older adults’ perception of their own risk of fall has never been included into screening tools. The goal of this study was to evaluate the predictive validity of questions on subjects’ self-perception of their own risk of fall.

**Methods:**

This prospective study was conducted on a probabilistic sample of 772 Spanish community-dwelling older adults, who were followed-up for a one year period. At a baseline visit, subjects were asked about their recent history of falls (question 1: “Have you fallen in the last 6 months?”), as well as on their perception of their own risk of fall by using two questions (question 2: “Do you think you may fall in the next few months?” possible answers: yes/no; question 3: “What is the probability that you fall in the next few months?” possible answers: low/intermediate/high). The follow-up consisted of quarterly telephone calls, where the number of falls occurred in that period was recorded.

**Results:**

A short questionnaire built with questions 1 and 3 showed 70% sensitivity (95% CI: 56%-84%), 72% specificity (95% CI: 68%-76%) and 0.74 area under the ROC curve (95% CI: 0.66–0.82) for prediction of repeated falls in the subsequent year.

**Conclusions:**

The estimation of one’s own risk of fall has predictive validity for the occurrence of repeated falls in older adults. A short questionnaire including a question on perception of one’s own risk of fall and a question on the recent history of falls had good predictive validity.

## Introduction

Falls may result in severe injury–including fracture–restriction of motility, functional deterioration and fear of falling and may be a determinant of institutionalization [1–3]. Approximately 10% of the older population experience repeated falls in one year with the consequent health risks [4]. Given that some risk factors may be modified and several interventions may be useful to reduce the risk of fall, it is important to have tools, which accurately evaluate such risk, predicting whether a subject will experience repeated falls in the future.

In the clinical practice, several tools are used to identify older people at risk of falling, to proceed with further evaluations and to intervene if necessary. Most of such tools and protocols are based on the analysis of subjects’ gait, balance and other functional mobility assessments; other tools include risk factors obtained from the past medical history of the patient, especially the history of previous falls [5,6]. Both approaches have certain limitations, which result in sub-optimal validity of the existing tools. Risk estimations based on subject’s functional mobility are restricted to the moment of examination and might lead to underestimation of the risk of fall in situations where the risk fluctuates (e.g. morning instability secondary to night sedatives) or where the risk comes from extrinsic factors (which are not present during examination or are unrelated to gait/balance). Risk estimations based on the history of falls would cover all circumstances–regardless of whether they were present during examination or not. However, they could be subjected to memory bias, since patients tend to forget falls, especially if they were inconsequential [7].

Here we propose the hypothesis that older adults are able to estimate well their own risk of fall and that such “self-estimation” could contribute to identification of subjects at risk of fall and injury. We postulate that perception of one's own risk of falling is the result of the action of different risk factors on the person. Thus, the overall self-perception of risk could be influenced by many other circumstances, difficult to detect individually. Inquiring about self-perceived risk, could yield different results than inquiring for the risk factors themselves. For example, a person who has forgotten that he has fallen could still retain a sense of risk, caused by those forgotten falls.

Our hypothesis has been previously tested in a small study, where self-perception of the risk of fall was predictive of subsequent falls in a cohort of older patients managed in our Geriatrics Service [8]. The goal of the present study was to evaluate the predictive validity of self-estimated risk of fall in a larger sample of community-dwelling subjects, as well as to evaluate the validity of an ultra-short questionnaire, which combined a question on self-perception and a question on the history of falls. In both cases, the evaluation of predictive validity was focused on the occurrence of multiple falls (not only one), because subjects who tend to fall repeatedly are at highest risk of complications.

## Materials and methods

This longitudinal, prospective study of predictive validity involved a cohort of 772 non-institutionalized, older-than-64-years subjects living in Spain. The study included a first (baseline) in-person visit and a subsequent one-year follow-up period based on quarterly telephone calls.

Subjects were recruited though a randomized multistage stratified sampling process according to sex, geographical area and community size (rural community, urban community, big city). The sample included a non-proportional age stratum with overrepresentation of subjects older than 79 years because, in our opinion, they are highly representative of the particular physiological characteristics of the elderly. In a first sampling stage, communities from pre-established geographical areas were selected, including different-sized communities in each area. Within communities, different districts were selected and, within every district, homes were selected by using a mixed door-to-door/telephone call approach. Information from the latest available register of inhabitants (2007) was used for the sampling process. Data were collected between years 2007 and 2009.

During the baseline visit, socio-demographic data (age, sex, education level, cohabitation status), a list of medicines and a list of previous diseases were collected by using a questionnaire from the National Health Survey 2006 [9]. The cognitive capacity was measured with the Pfeiffer’s test [10,11] and the independence in activities of daily living (ADL) was evaluated with the Katz’s index [12]. Depression was screened by using the GDS-5 [13], muscle strength in lower extremities was assessed by using the Medical Research Council Scale [14], and balance was measured by using the following 4 items of the Tinetti Balance Scale: sitting balance, attempts to rise (stand up), immediate standing balance and standing balance [15].

The occurrence of falls in the previous 6 months was investigated by using the question: “Have you fallen in the last 6 months?” (Question 1; possible answers: yes/no). The self-perception of the risk of fall was evaluated by using two questions [8]: “Do you think you may fall in the next few months?” (*¿Cree que se puede caer en los próximos meses*?) (Question 2; possible answers: yes/no) and “What is the probability that you fall in the next few months?” (*¿Cuál es la probabilidad de que se caiga en los próximos meses*?) (Question 3; possible answers: low/intermediate/high). If a participant asked exactly how many months, the survey taker asked for a 3-month prediction. Question 1 could be answered by the subject or by an accompanying person. Questions 2 and 3 had to be answered by the subject. Additionally, we combined answers to question 1 and question 3 in a scale which ranks from 1 through 6, according to the scoring system depicted in Table 1.

**Table 1:** Scoring of a questionnaire built with two questions regarding history of falls and perceived fall risk.

Previous falls (6 months)	Perceived risk of falling	SCORE
No	Low	1
	Medium	2
	High	3
Yes	Low	4
	Medium	5
	High	6

Questions for score calculation: 1.- Have you ever fallen in the last 6 months?; 2.- What is the probability that you fall in the next few months?

In the follow-up telephone calls, the number of falls experienced by the subject from the baseline visit or the previous telephone call was recorded. A fall was defined as any event, where the subject unintentionally came to rest on the ground or a lower level. However, this working definition was not provided to patients, but instead the more familiar term “fall”, included in direct simple questions, was used throughout the interview (e.g. “Have you fallen down since the last telephone call?”). On the basis of the subjects’ answers, the survey taker decided whether an event had been a fall or not.

Baseline data were recorded by professional survey takers without specific training in healthcare. In order to reduce non-sampling errors, the interview was carefully designed, the survey takers were trained and the field-work, surveys, data-coding and data processing were carefully supervised. All survey takers received the same training, which consisted of theoretical and practical sessions on the administration of the survey and the tools included in it. In order to minimize the lack of response, a candidate was replaced with another after 10 failed attempts to contact, subject’s failure to attend 2 appointments, refusal to participate, institutionalization or death. The final response rate was 63%. To ensure the quality of the field-work a supervisor accompanied the survey takers during the first few visits and a telephonic control was carried out on 15% of participants; they were asked about the sampling procedure (how they had been contacted), the completion of the protocol and the veracity of answers. The fulfilled questionnaires were reviewed by a specially constituted team, which was also in charge of recovering missing data by telephone, whenever possible. Such quality control revealed selection errors for 100 participants, who were not included in the cohort.

Follow-up data were collected by telephone interviews 4, 6, 9 and 12 months after the baseline visit. The people in charge of the follow-up used a structured interview model. They had received theoretical and practical training in the administration of the interviews. The interviewers were blind to the participant's answers to the tested questions.

The research protocol was previously approved by the local Ethical Committee (Consorci Sanitari del Maresme). All participants had to sign an informed consent form before being included in the study.

### Statistical analysis

To avoid overrepresentation of any age or sex group, the sample was weighted according to the population in the 2012 annual report of the Spanish National Institute of Statistics (INE) and stratified by sex and age groups (65-to-79 and older-than-79). Subjects with cognitive deterioration (Pfeiffer test > 7) were excluded from the analysis. Hospitalization, institutionalization or variations from baseline in the following risk factors lead to exclusion from subsequent follow-up (end of follow-up): initiation or termination of any medication that increased the risk of fall (benzodiazepines, antidepressants, neuroleptics, digoxin and antiarrhythmics A1c) or initiation or termination of physiotherapy or occupational therapy aimed at reducing the risk of fall. The reason for such exclusion criteria was that the self-perception of the risk of fall–which was only recorded at baseline–could vary throughout the follow-up in case these factors substantially modified the risk.

Sensitivity, specificity and area under the ROC curve (AUC) were calculated for the studied questions (questions 1, 2 and 3), as well as the Odds Ratio (OR) and Relative Risk (RR) associated to positive responses. The short questionnaire built with questions 1 and 3 was studied in the same way. The occurrence of falls during follow-up (gold standard), was considered positive when more than one fall was reported in a certain period (repeated falls), and negative, when no falls or a single fall occurred. Patients with missing data were scarce; these patients were excluded from the specific analysis affected by data-missing.

A 583 subject sample was considered sufficient for a 0.6 expected AUC with 95% confidence and 0.08 estimation precision, considering a 1/10 proportion of participants with repeated falls [16]

Calculations were carried out with the “survey” software package R v.3.1.2 [17,18]

## Results

This study included 772 subjects, 550 of them older than 80 years; 637 participants completed the follow-up, 430 of them were octogenarian (17% were lost to follow-up); 9.9% of participants had multiple falls during the one-year follow up period, the incidence of falls in this sample was published elsewhere [19]. Twenty subjects were excluded from the analysis due to severe cognitive deterioration (Pfeiffer test > 7 errors). Furthermore, 292 subjects were excluded during follow-up due to changes in medication (which increased the risk of fall), initiation or termination of rehabilitation therapy (which modified the risk of fall), hospitalization, institutionalization or death.

**Fig 1:**
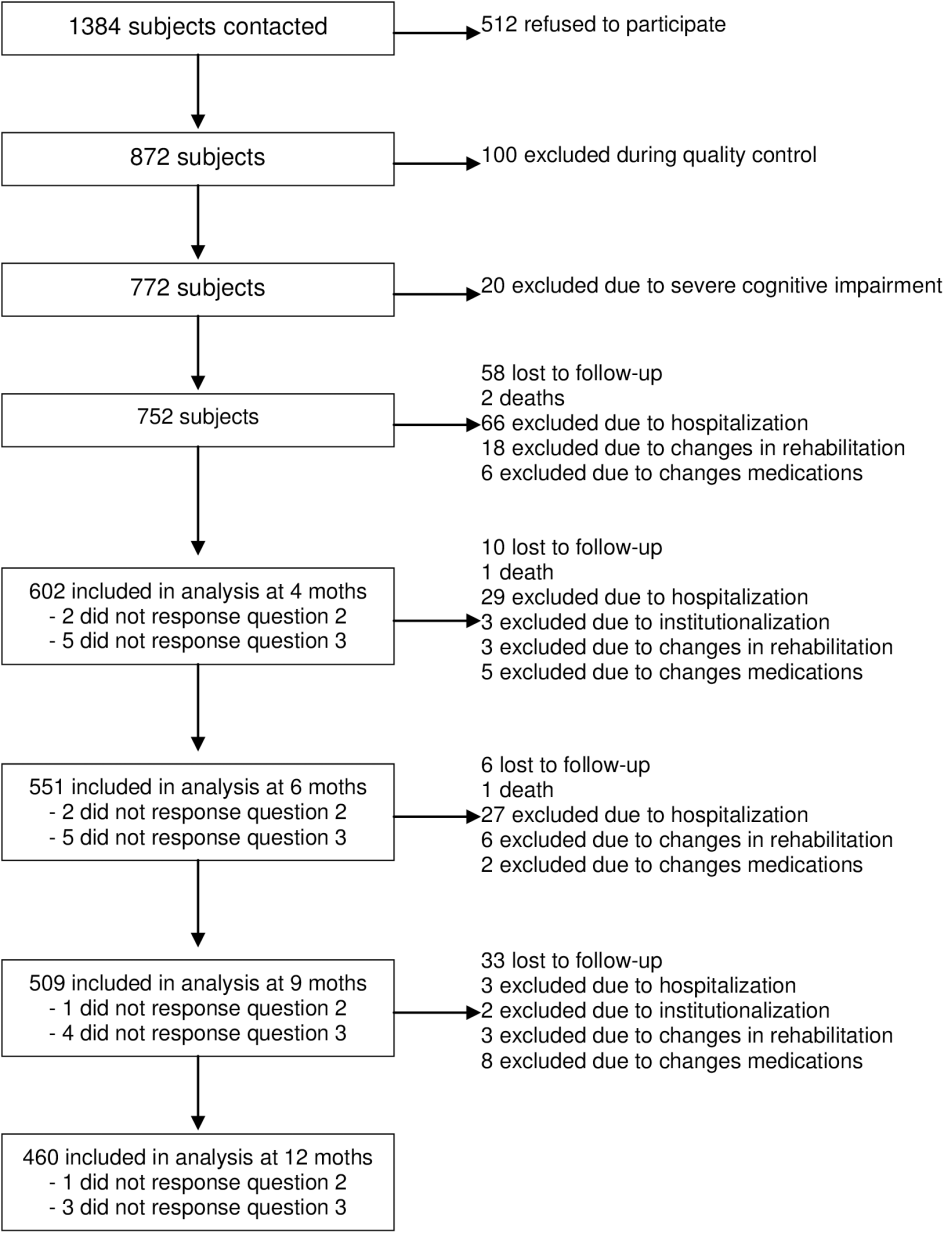
Subject exclusion and loss during the study.

Thus, by the end of the one-year follow-up, complete datasets had been obtained for 460 subjects (61.17%). Fig 1 shows detailed information on subjects lost to follow-up in each period. The median follow-up time was 10.66 months (SD 2.62). Table 2 shows the socio-demographic data of the 602 subjects, who completed at least one telephonic control, and their answers to the tested questions.

**Table 2:** Socio-demographic data of participants who completed the first follow-up period.

Age	Median	SD
	80.7	0.1
**Sex**	**n**	**%**
Male	226	37.5
Female	376	62.5
**Education level**	**n**	**%**
None	165	27.8
Basic	337	56.7
Intermediate	59	9.9
University	32	5.4
**Cohabitation**	**n**	**%**
Living alone	162	27.4
Cohabitating with someone	430	72.6
**Katz**	**n**	**%**
0	397	66.0
1	112	18.6
2	21	3.5
3	17	2.8
4	12	2.0
5	21	3.5
6	22	3.7
**Pfeiffer**	**n**	**%**
0–3	524	88.0
4–7	71	12.0
8–10	Excluded	Excluded
**Tested questions** ^**a**^	**n**	**%**
Question 1: affirmative answer	159	26.4
Question 2: affirmative answer	197	32.9
Question 3: intermediate risk	221	37.2
Question 3: high risk	66	11.1

a Tested questions: Question 1: Have you fallen in the last 6 months? A: Yes vs. No; Question 2: Do you think you may fall in the next few months? A: Yes vs. No; Question 3: What is the probability that you fall in the next few months? A: low / intermediate / high.

Table 3 shows the RR for repeated falls (two or more) corresponding to the three questions at every follow-up point, as well as the sensitivity and specificity of every question for the prediction of falls.

**Table 3:** Predictive validity of every one of the questions in the study for the occurrence of repeated falls.

		month 4	month 6	month 9	month 12
		Central (95% CI)	Central (95% CI)	Central (95% CI)	Central (95% CI)
**Sensitivity (%)**	Question 1	63 (33–86)	55 (32–76)	**57 (37–75)**	**64 (44–80)**
	Question 2	**78 (46–94)**	**60 (37–80)**	50 (31–70)	51 (32–69)
	Question 3	68 (36–89)	54 (32–75)	56 (36–75)	63 (42–80)
**Specificity (%)**	Question 1	**78 (74–82)**	**79 (74–83)**	**79 (74–83)**	**79 (74–84)**
	Question 2	69 (64–73)	70 (64–75)	70 (64–75)	69 (63–75)
	Question 3	63 (58–69)	64 (59–69)	64 (59–69)	64 (58–70)
**Odds Ratio**	Question 1	6.2 (1.6–23.5)	**4.6 (1.7–12.6)**	**5.1 (2.1–12.3)**	**6.6 (2.7–16.2)**
	Question 2	**7.9 (1.7–36.3)**	3.5 (1.7–9.6)	2.4 (1.0–5.6)	2.3 (1.0–5.4)
	Question 3	3.7 (0.9–15.3)	2.2 (0.8–5.9)	2.3 (1.0–5.5)	3.0 (1.3–7.3)
**Relative Risk**	Question 1	5.8 (1.6–12.7)	**4.2 (1.7–7.3)**	**4.4 (2.1–6.7)**	**5.5 (2.6–7.9)**
	Question 2	**7.4 (1.7–16.5)**	3.2 (1.3–6.2)	2.2 (1.0–3.9)	2.2 (1.0–3.9)
	Question 3	3.6 (0.9–9.2)	2.1 (0.8–4.3)	2.2 (1.0–3.9)	2.7 (1.3–4.7)

Question 1: Have you fallen in the last 6 months? A: Yes vs. No; Question 2: Do you think you may fall in the next few months? A: Yes vs. No; Question 3: What is the probability that you fall in the next few months? Answers clustered into two categories low vs. intermediate/high.; Reference group for calculations: participants with no falls or a single fall. Maximum values in bold type.

The short questionnaire (built with questions 1 and 3) yielded scores between 1 and 6. The proportion of subjects who suffered repeated falls during the one-year follow-up was found to increase with the score; thus, it was lowest for subjects with score 1 and highest for those with score 6. Establishing score 3 as a cut point, the sensitivity and specificity for the occurrence of repeated falls in one year were: 70% (95% CI 56%–84%) and 72% (95% CI 0.68%–0.76%), respectively. Table 4 shows the incidence of repeated-falls for every score (range 1 to 6), as well as the sensitivity and specificity for every cut point. The area under the ROC curve for the different scores from the questionnaire for prediction of repeated falls in one year was 0.74 (95% CI: 0.66–0.82). Table 5 shows the area under the ROC curve for the questionnaire and for questions 1, 2 and 3 in every follow-up period. The area under the ROC curve of the questionnaire for the prediction of repeated falls (one year) in the oldest group was 0.72 (95%CI: 0.62–0.81) and in the under 80 year group was: 0.77 (95%CI: 0.60–0.93), not being the difference significant among groups (p = 0.6). Also, not statistical differences were found in questionnaire validity at one year among sex groups: women 0.68 (95%CI: 0.58–0.78); men 0.84 (95%CI: 0.70–0.97); p = 0.07

**Table 4:** Participants with repeated falls in every control period, according to their score in a questionnaire based on previous falls and self-estimated probability of fall (scores 1 to 6).

SCORE	MONTH 4			MONTH 6			MONTH 9			MONTH 12		
	% of fallers^a^ (95% CI)	Sensitivity (%)	Specificity (%)	% of fallers^a^ (95% CI)	Sensitivity (%)	Specificity (%)	% of fallers^a^ (95% CI)	Sensitivity (%)	Specificity (%)	% of fallers^a^ (95% CI)	Sensitivity (%)	Specificity (%)
**1**	0.89 (0.17–4.61)	100	0	2.62 (1.00–6.66)	100	0	3.73 (1.63–8.31)	100	0	2.64 (1.00–6.81)	100	0
**2**	1.12 (0.36–3.46)	87	45	2.82 (0.67–11.10)	78	45	3.36 (0.93–11.37)	79	45	4.61 (1.61–12.56)	84	45
**3**	8.03 (1.18–38.85)	67	70	8.88 (1.31–41.66)	67	71	9.4 (1.39–43.36)	68	71	10.84 (1.60–47.60)	**70**	**72**
**4**	4.66 (0.9–20.82)	60	75	9.95 (3.13–27.44)	63	75	11.39 (3.87–29.12)	66	76	14.4 (5.52–32.64)	67	77
**5**	8.43 (2.68–23.57)	45	83	12.01 (4.95–26.35)	48	83	17.79 (8.50–33.50)	53	84	19.83 (9.96–35.59)	51	84
**6**	14.25 (3.61–42.46)	20	94	21.79 (7.56–48.70)	19	95	32.44 (15.32–56.02)	84	95	**34.16** **(16.17–58.27)**	21	95

a. Fallers: subjects with two or more falls in every follow-up period. Non Fallers: people with no falls or a single fall. Maximum values in bold type

**Table 5:** Area under the ROC curve for every studied question and for the questionnaire, at every follow-up period.

Area under the curve	month 4	month 6	month 9	month 12
Question 1^**a**^	0.68 (0.55–0.81)	0.69 (0.60–0.78)	0.71 (0.63–0.79)	0.72 (0.65–0.79)
Question 2^**a**^	0.71 (0.59–0.82)	0.66 (0.57–0.75)	0.61 (0.53–0.69)	0.56 (0.48–0.64)
Question 3^**a**^	0.66 (0.52–0.79)	0.60 (0.49–0.71)	0.62 (0.53–0.71)	0.62 (0.54–0.70)
Questionnaire*	**0.73 (0.60–0.86)**	**0.70 (0.59–0.81)**	**0.72 (0.63–0.82)**	**0.74 (0.66–0.82)**

Question 1: Have you fallen in the last 6 months? A: Yes vs. No; Question 2: Do you think you may fall in the next few months? A: Yes vs. No; Question 3: What is the probability that you fall in the next few months? A: low / intermediate / high.; Questionnaire: includes questions 1 and 3

The other measured risk factors for falling presented the following areas under the ROC curve, for the prediction of repeated falls in one year: Polypharmacy: 0.67 (95% CI: 0.58; 0.77); muscle weakness: 0.50 (95% CI: 0.41; 0.60); impaired balance: 0.61 (95% CI: 0.51; 0.70); functional impairment 0.54 (95% CI: 0.45; 0.62); depression 0.63 (95% CI: 0.54; 0.72); cognitive impairment 0.52 (95%CI: 0.43; 0.61).

## Discussion

Up to our knowledge, the self-perceived high risk of fall has never been considered as a risk marker or included in screening tools. The results of this study showed however, that older adults’ self-estimation of their own risk of fall had a strong predictive value for the incidence of subsequent falls. It was clear that subjects, who believed that they were at risk of falling, experienced more falls than others. Question 2 (Do you think you may fall in the next few months?) had good validity to detect subjects prone to repeated falls in the short and medium term (up to 6 months). In the long term (6 to 12 months), experiencing at least one fall in the previous 6 months had higher predictive capacity. The combination of the history of falls and the self-perception of one’s own risk–as collected by the described questionnaire–had very good predictive capacity at any time, up to one year. Additionally, this 6-score-questionnaire accurately stratified subjects into risk groups, where the incidence of falls was directly proportional to the questionnaire score at any moment in time. It is worth noting that more than one-third of subjects with score 6 experienced repeated falls in the next year, while subjects with scores 1 and 2 experienced less repeated falls than the general population (incidence about 10%) [20–22]. We would like to highlight that this questionnaire can be administered in a short time and that it can be used even with subjects, who have moderate cognitive deterioration (Pfeiffer <8) and that there are no significant differences in its validity among different age or sex groups. Finally, it is important to mention that the AUC of the questionnaire is higher than that of any other risk factor for falls analyzed in our study.

Few reports were found in the literature on the predictive validity of the most extensively used tools to assess the risk of fall. They were often studied through convergent validity, by comparing with other available tools for estimation of the risk of fall, or through case-control studies where the gold standard was retrospective. One of the tools, for which predictive validity was studied, was the Performance-Oriented Mobility Assessment (POMA) scale, by Tinetti. Verghese et al. found 61.5% sensitivity and 69.5% specificity in a group of 60 community-dwelling older people, for the balance sub-scale by using a cut point of 10 [23]. Faber et al. found, in a group of 72 institutionalized and community-dwelling older adults, that the sensitivity and specificity to detect “fallers” (more than one fall) varied between 62.5% and 66.1%, even with the best cut point [24]. Lin et al. studied the predictive validity of the *Timed Up and Go (TUG)*, *One-Leg Stand*, *Functional Reach (FR)*, and *Tinetti Balance scale* (POMA) on a sample of community-dwelling older adults in Taiwan [25]. In this study, TUG presented a 0.61 AUC for prediction of falls in the following year, while the AUC of the other tests was between 0.51 and 0.56. However, these authors did not measure the tests’ predictive capacity for recurrent falls so that their results are not fully comparable to ours. Russell et al. studied the predictive validity of FR and TUG on a sample of Australian older adults, who attended the hospital because they had suffered a fall and were followed-up for 12 months. The area under the curve for prediction of further falls was 0.60 for FR and 0.63 for TUG. The area under the curve for recurrent falls was 0.62 for both tests, namely lower than that of our questionnaire. In the same study, the authors reported a slightly higher validity for the *Falls risk for older people in the community* (FROP-Com) tool for assessment of recurrent falls, although it was also lower than that of our short questionnaire (AUC 0.68) [26]. Thus, we failed to find any other tool for prediction of the risk of fall with better validity than ours, in a prospective study.

Identifying subjects prone to recurrent falls is of high clinical interest because they often experience severe complications of falls. In our opinion, older people who estimate their probability of falling to be “high” (according to question n°3) or who have experienced at least one fall in the last 6 months (question n°1) should be evaluated with the aim of reducing their risk. Any one of the two circumstances scores a minimum of 3 in our short questionnaire. This is also valid for subjects who answer “yes” to question 2: “Do you think you may fall in the next few months?”

In addition, falls are related to fragility syndrome, which is a lack of functional reserve, predisposing to adverse health events such as falls, functional deterioration of death [27]. Thus, the development of an instrument that quantifies the risk of falling may be useful to quantify fragility or at least one of its components. It is likely that patients who score higher on the instrument are more fragile. In line with our research, a recently published study shows how self-reported information on the components of fragility, had a superior discriminatory and predictive capacity compared to the Fried fragility phenotype. In that study, a model which included self-reported measures of slow walking, low physical activity and weight loss, had a higher AUC (0.64) for incident falls compared to the Fried fragility phenotype (AUC: 0.57) and the FRAIL scale (AUC: 0.56) [28]. Therefore, self-reported falls or self-reported risk for falling may also contribute to improve frailty detection.

Our study had several limitations that are worth commenting. The self-estimation of the risk of fall was recorded at the beginning of the study but not during the follow-up period. Modifications in the risk of fall during follow-up could be accompanied by corresponding changes in self-estimated risk of fall, which we did not measure. Therefore, subjects with changes in medication or therapies that could modify the risk of fall were excluded from the analysis. This resulted in a substantial reduction of the sample, which may be detrimental to the generalization of results. Furthermore, we made quarterly telephone calls (except for the first one, which was made 4 months after the baseline visit). Thus, there was time enough for memory bias between two consecutive evaluations, which would result in a tendency to underestimate the incidence of falls [7]. However, we consider that such a bias was not important in our study because the global incidence of falls and recurrent falls in our sample–published elsewhere–were similar to those of other studies [19].

In conclusion, older adults’ perception of their own risk of fall is valid as a predictor of recurrent falls. A short questionnaire including a question on self-perception of one’s own risk of fall and a question on the occurrence of falls in the previous 6 months shows good predictive validity for the occurrence of multiple falls in one year.

## Supporting information

S1 Tool Screening instrument for recurrent falls. (TIF)
