# Supplementary figures and images for: A two-question tool to assess the risk of repeated falls in the elderly
Source: PLoS One. 2017 May 10;12(5):e0176703. doi: 10.1371/journal.pone.0176703 (PMC5425174; doi:10.1371/journal.pone.0176703)

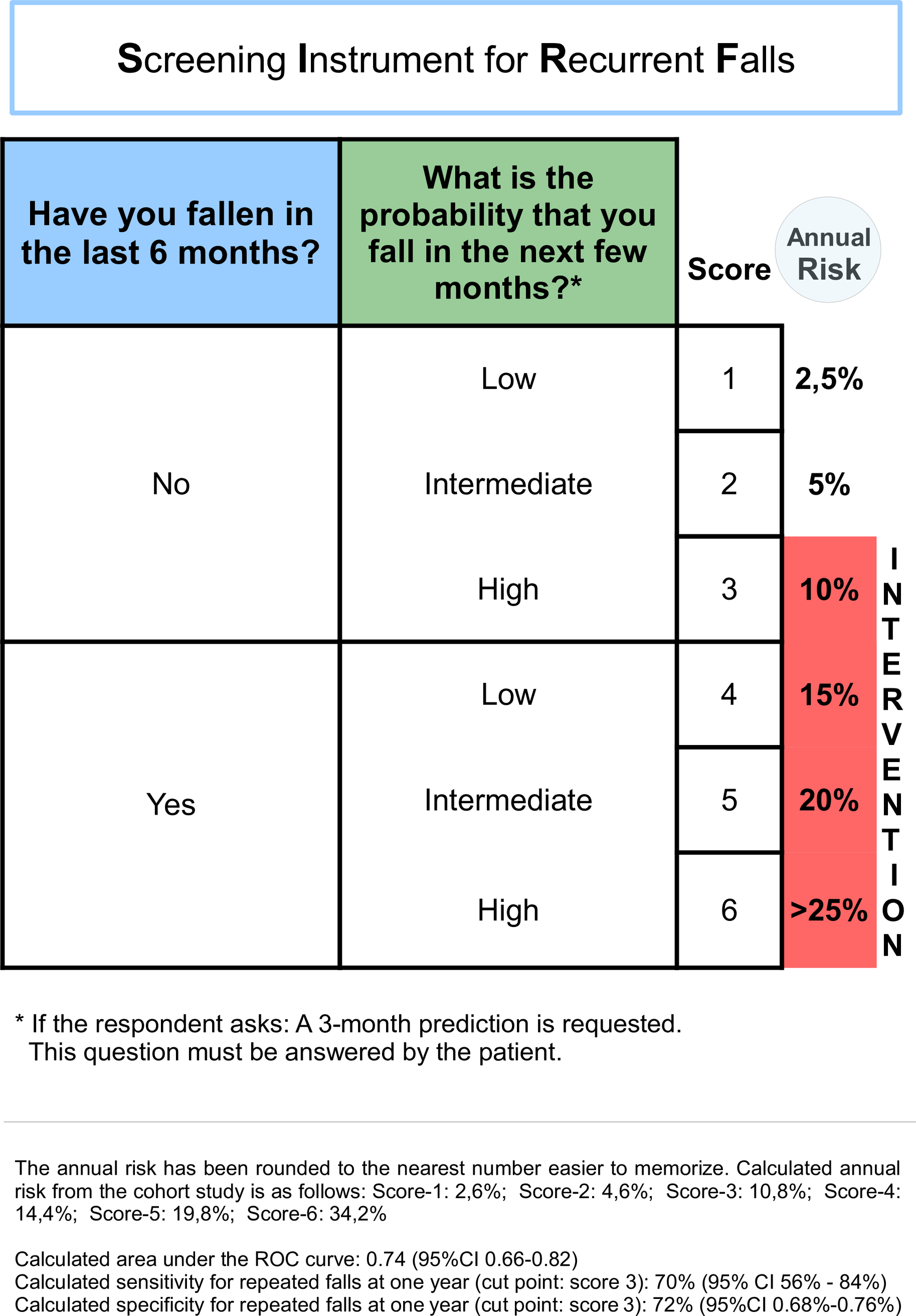

Supplement: S1 Tool — (TIF) [file pone.0176703.s001.tif]
